# Author Correction: Apolipoprotein J is a hepatokine regulating muscle glucose metabolism and insulin sensitivity

**DOI:** 10.1038/s41467-020-16305-6

**Published:** 2020-05-05

**Authors:** Ji A. Seo, Min-Cheol Kang, Won-Mo Yang, Won Min Hwang, Sang Soo Kim, Soo Hyun Hong, Jee-In Heo, Achana Vijyakumar, Leandro Pereira de Moura, Aykut Uner, Hu Huang, Seung Hwan Lee, Inês S. Lima, Kyong Soo Park, Min Seon Kim, Yossi Dagon, Thomas E. Willnow, Vanita Aroda, Theodore P. Ciaraldi, Robert R. Henry, Young-Bum Kim

**Affiliations:** 1 Division of Endocrinology, Diabetes, and Metabolism, Beth Israel Deaconess Medical Center and Harvard Medical School, Boston, MA USA; 2 Division of Endocrinology, Department of Internal Medicine, Korea University College of Medicine, Seoul, Korea; 3 Research Group of Food Processing, Korea Food Research Institute, Wanju-gun, Jeollabuk-do Korea; 4 Department of Molecular Medicine and Biopharmaceutical Sciences, Graduate School of Convergence Science and Technology, Seoul National University, Seoul, Korea; 5 Division of Nephrology, Department of Internal Medicine, College of Medicine, Konyang University, Daejeon, Korea; 6 Department of Internal Medicine and Biomedical Research Institute, Pusan National University Hospital, Busan, Korea; 7 Department of Internal Medicine, Asan Medical Center, University of Ulsan, College of Medicine, Seoul, Korea; 8 Molecular Cardiovascular Research, Max-Delbrueck-Center for Molecular Medicine, Berlin, Germany; 9 Veterans Affairs San Diego Healthcare System (9111 G), San Diego, CA 92161 USA; 10 Department of Medicine, University of California San Diego, La Jolla, CA 92093 USA; 11 Present Address: Columbia University, New York, NY USA; 12 Present Address: School of Applied Science, University of Campinas, Limeira, Brazil; 13 Present Address: East Carolina University, East Carolina Diabetes and Obesity Institute, Greenville, NC USA; 14 Present Address: College of Medicine, The Catholic University of Korea, Seoul, Korea; 15 Present Address: Universidade Nova de Lisboa, Lisboa, Portugal; 16 Present Address: Brigham and Women’s Hospital and Harvard Medical School, Boston, MA USA

**Keywords:** Metabolism, Endocrine system and metabolic diseases

Correction to: *Nature Communications* 10.1038/s41467-020-15963-w, published online 24 April 2020.

The original version of this Article contained errors in the author affiliations.

Kyong Soo Park and Young-Bum Kim were incorrectly associated with the Research Group of Food Processing, Korea Food Research Institute, Wanju-gun, Jeollabuk-do, Korea.

Kyong Soo Park was associated with Department of Molecular Medicine and Biopharmaceutical Sciences, Graduate School of Convergence Science and Technology, Seoul National University, Seoul Korea.

This has now been corrected in both the PDF and HTML versions of the Article.

