# Supplementary material for: Apolipoprotein J is a hepatokine regulating muscle glucose metabolism and insulin sensitivity
Source: Nat Commun. 2020 Apr 24;11:2024. doi: 10.1038/s41467-020-15963-w (PMC7181874; doi:10.1038/s41467-020-15963-w)
Supplement: Supplementary file 1 — Supplementary Information [file 41467_2020_15963_MOESM1_ESM.pdf]

Supplementary information

ApolipoproteinJ is a hepatokine regulating muscle glucose metabolism and insulin sensitivity

Seo et al.

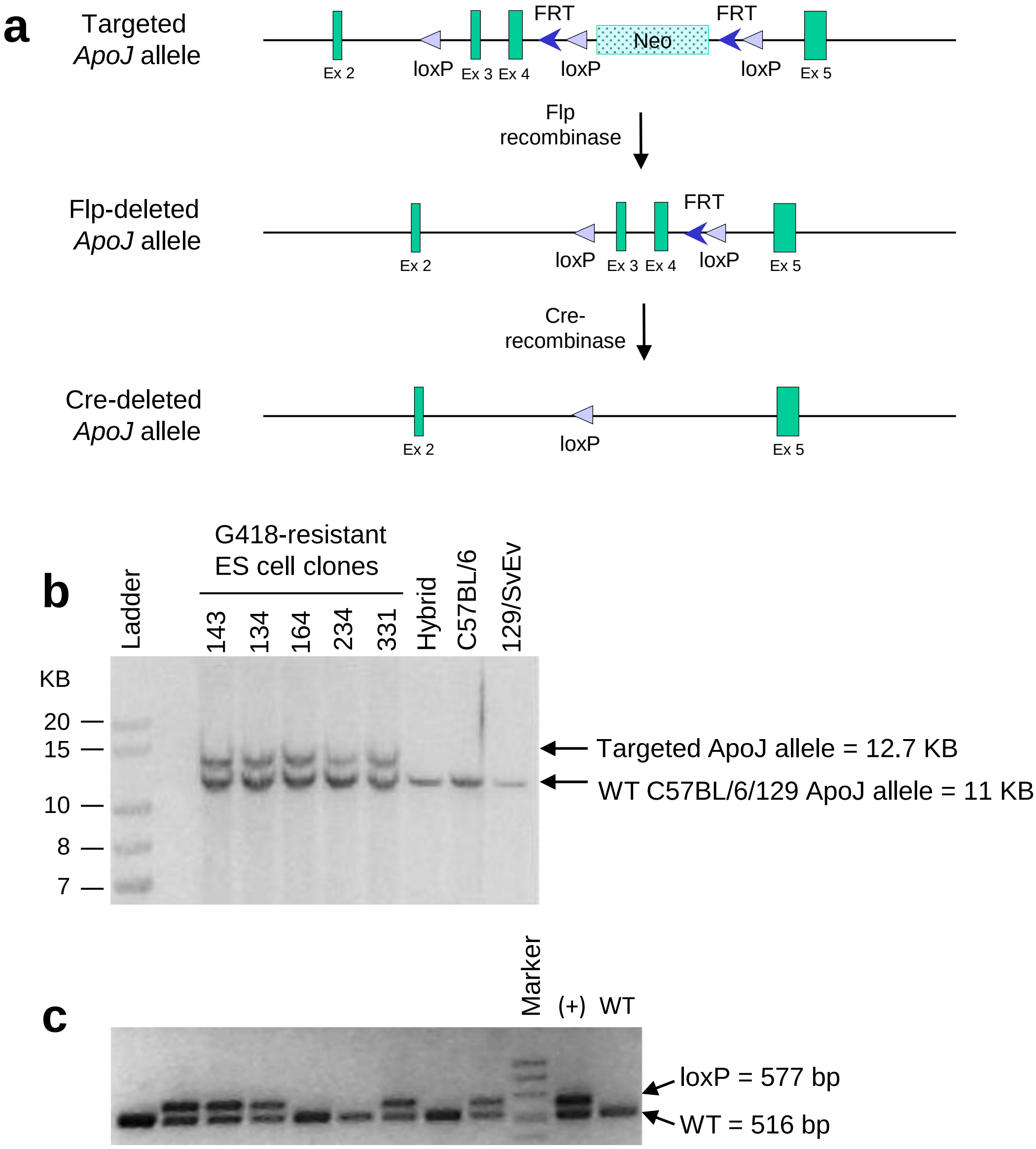

Supplementary Fig. 1 Generation of ApoJ$^{loxP/loxP}$ mice.

(a) Schematic representation of wild type ApoJ allele, targeting vector, targeted allele and flox allele. The targeting vector containing loxP sites flanking exon 3-4 of ApoJ was injected into embryonic stem cells. (b) Southern blot analysis of G418 resistant ES cells. DNA encoding the ApoJ targeting vector was linearized by NsiI and then transfected by electroporation of iTL BA1 (C57BL/6 x 129/SvEv) hybrid embryonic stem cells. After selection in G418 antibiotic, surviving clones were expanded for Southern analysis to identify recombinant ES clones. DNA was digested with Pst1. iTL BA1 ES cells contain one ApoJ allele from C57BL/6 mice and the other from 129 mice. ES cell DNA shows the WT C57BL/6/129 allele a band of 11 kb and the ApoJ targeted C57BL/6 allele which appears as a band shift from 11 to 12.7 kb. These data are representative from two independent experiments. (c) Genotyping of ApoJ loxP/loxP mice. Tail DNA samples from F1 mice were amplified by PCR using genomic primer pair, which flanks one of loxP sites. With the addition of loxP site, the amplified product is 577 bp. For wild-type (or without a loxP site), the amplified product is 516 bp. The positive control, indicated by plus, is positive ES clone. These data are representative from two independent experiments.

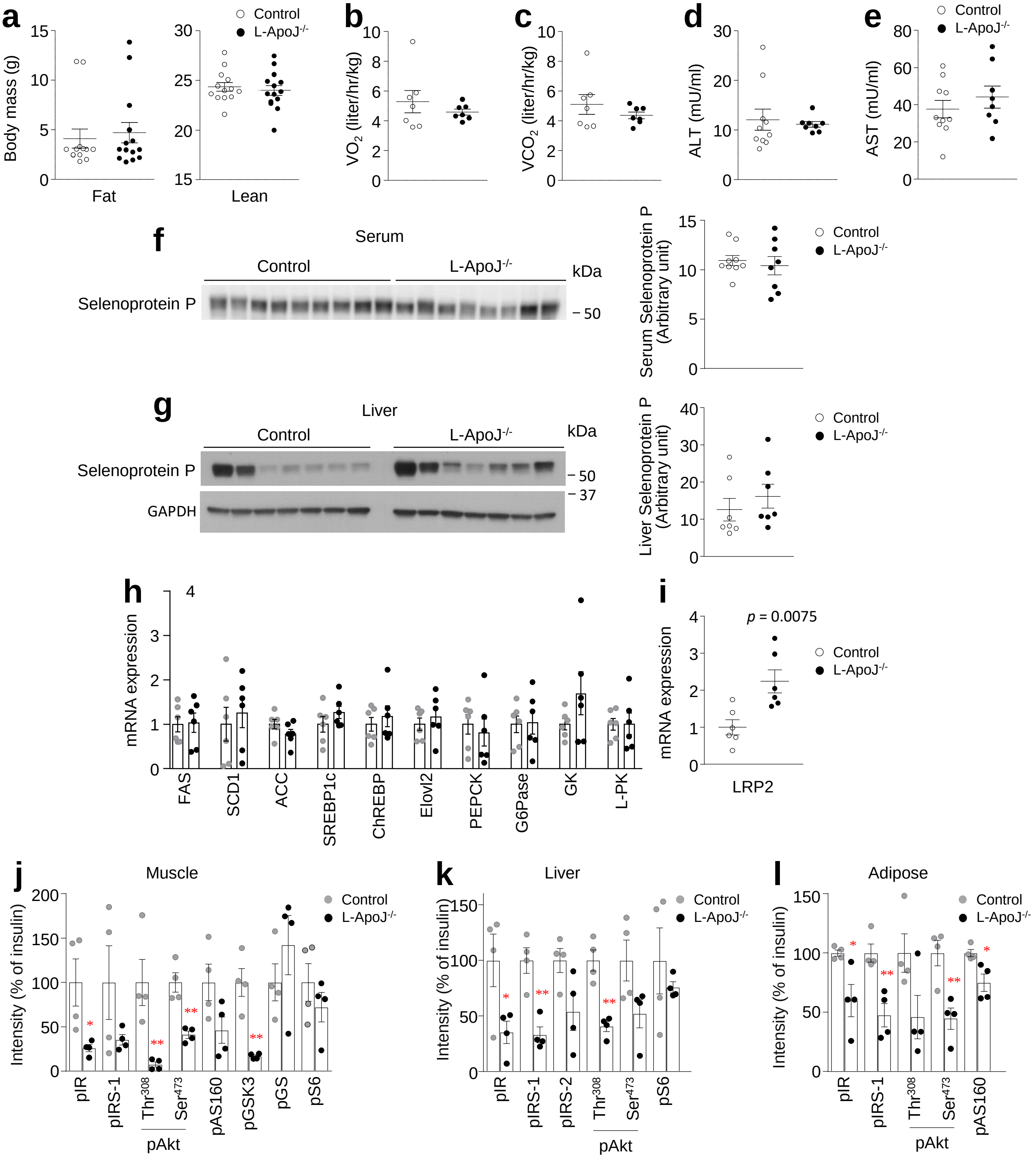

Supplementary Fig. 2 Loss of hepatic ApoJ decreases insulin signaling independent of adiposity.
(a) Body mass (n = 13 for control, n = 14 for L-ApoJ$^{-/-}$), (b) O_2 consumption (n = 7 per group), (c) CO_2 production (n = 7 per group), (d) serum ALT (n = 10 for control, n = 8 for L-ApoJ$^{-/-}$), (e) serum AST (n = 10 for control, n = 8 for L-ApoJ$^{-/-}$), (f) serum Selenoprotein P (n = 9 for control, n = 8 for L-ApoJ$^{-/-}$), (g) liver Selenoprotein P (n = 7 per group), (h) hepatic gene expression (n = 6 per group), and (i) muscle LRP2 gene expression (n = 6 per group) in ApoJ$^{loxP/loxP}$ (Control) and liver-specific ApoJ-deficient mice (L-ApoJ$^{-/-}$) mice fed a chow diet. Body mass was measured by an MRI at 18-20 weeks of age. VO_2 and VCO_2 were measured by a comprehensive lab animal monitoring system (CLAMS) at 8 weeks of age. Serum parameters, liver Selenoprotein P levels, and LRP2 mRNA levels were measured from overnight fasted mice at 13−14 weeks of age. (j) Quantification of muscle insulin signaling components from Fig. 4p (n = 4 per group). (k) Quantification of muscle insulin signaling components from Fig. 4q (n = 4 per group). (l) Quantification of muscle insulin signaling components from Fig. 4r (n = 4 per group). All bars and errors represent means ± SEM. *p < 0.05, **p < 0.01 vs. control by two-sided Student's t test.

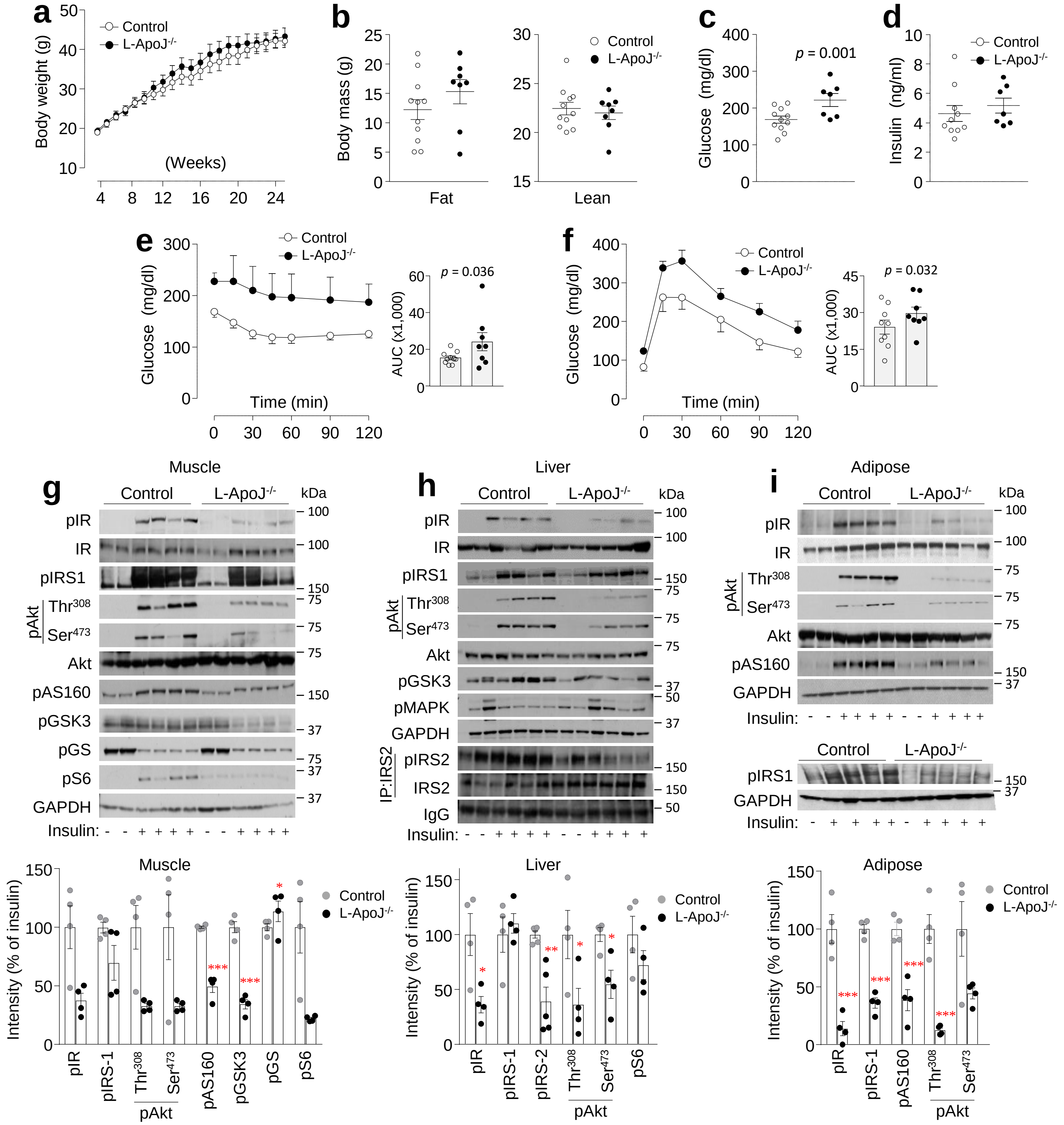

Supplementary Fig. 3 Mice lacking hepatic ApoJ develop insulin resistance and glucose tolerance under high-fat feeding.
(a) Body weight (n = 12 for control, n = 8 for L-ApoJ$^{-/-}$), (b) body mass (n = 11 for control, n = 8 for L-ApoJ$^{-/-}$), (c) blood glucose (n = 11 for control, n = 7 for L-ApoJ$^{-/-}$), (d) serum insulin (n = 10 for control, n = 7 for L-ApoJ$^{-/-}$), (e) insulin tolerance test (ITT) (n = 10 for control, n = 8 for L-ApoJ$^{-/-}$), and (f) glucose tolerance test (GTT) (n = 9 for control, n = 8 for L-ApoJ$^{-/-}$) in control and L-ApoJ$^{-/-}$ mice fed a high-fat diet (HFD). Blood glucose and serum insulin were measured in the fasting state at 14-18 weeks of age. Body mass was measured at 17 weeks of age. For ITT, mice were injected intraperitoneally with insulin at 0.75 U/kg of body weight. A GTT was performed in overnight fasted mice with an intraperitoneal injection of glucose at 1.0 g/kg of body weight. ITT was performed at 19 weeks of age and GTT was performed at 14 weeks of age. (g-i) Insulin signaling in muscle, liver, and adipose of control and L-ApoJ$^{-/-}$ mice fed a HFD. L-ApoJ$^{-/-}$ mice fed a HFD were injected intraperitoneally with human insulin (10 U/kg of body weight) or saline and scarified 10 min later at 24 weeks of age (n = 4 per group, n = 5 for liver pIRS2 per group). Phosphorylation of insulin signaling components were measured by immunoblotting or immunoprecipitation followed by immunoblotting, as indicated. Bars show densitometric quantitation of insulin signaling components from each tissue. AUC: Area under the curve. p values for AUCs were evaluated by one-sided Student's t-test. All bars and errors represent means ± SEM. $*p < 0.05$, $**p < 0.01$, $***p < 0.001$ vs. control by two-sided Student's t test.

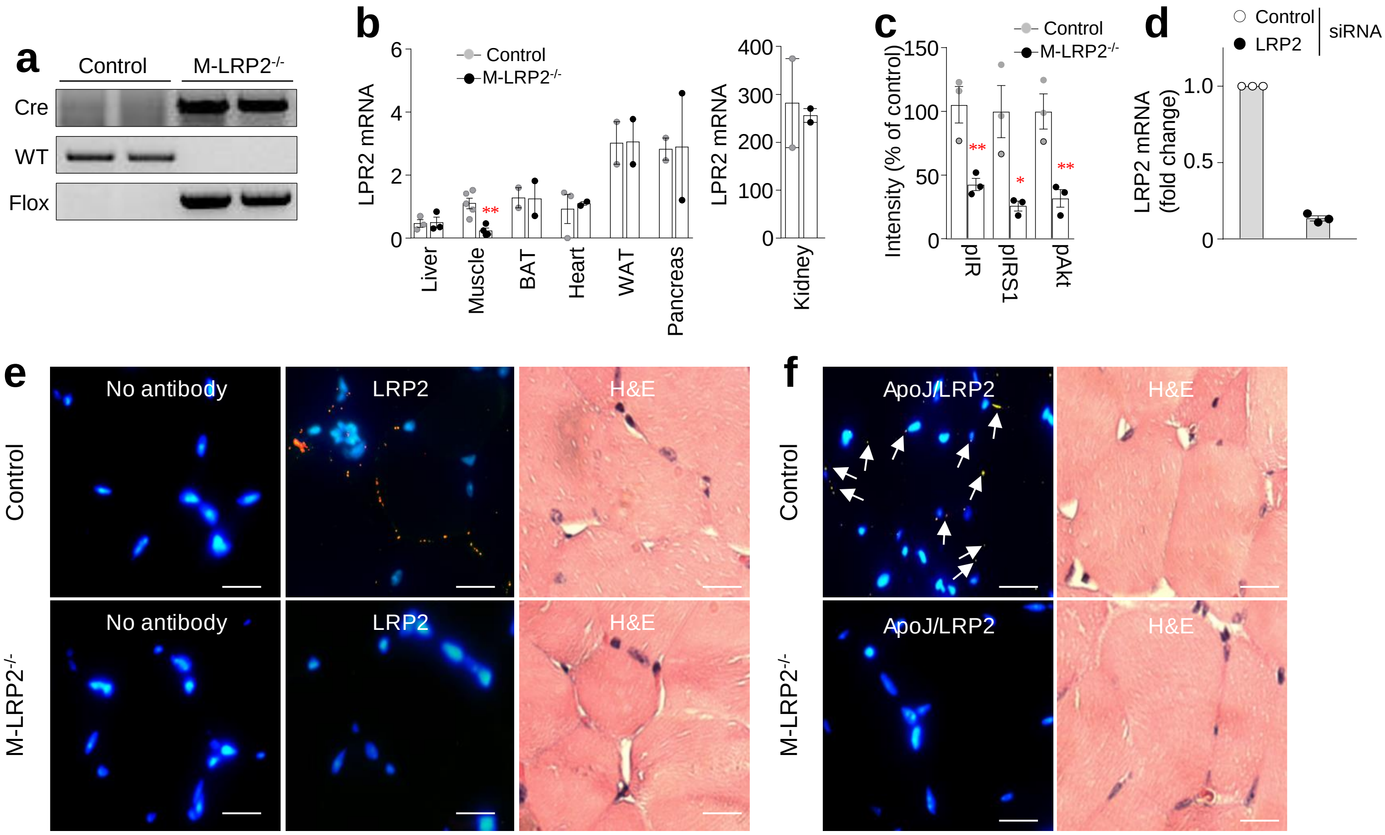

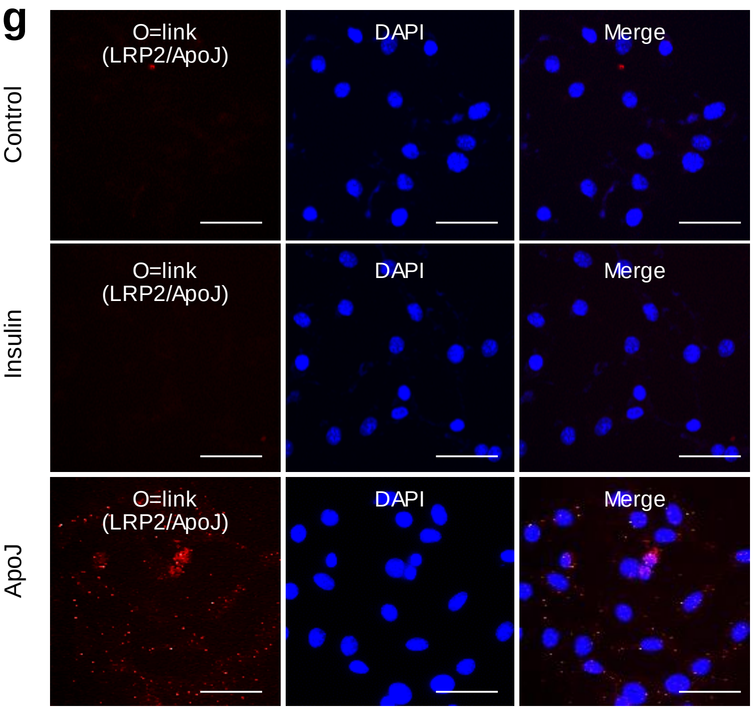

Supplementary Fig. 4 Deletion of muscle LRP2 impairs insulin signaling in muscle but not liver and adipose tissue. (a) Genotyping of muscle-specific LRP2-deficient mice (M-LRP2$^{-/-}$). M-LRP2$^{-/-}$ mice were generated by crossing mice with a floxed LRP2 allele to myogenin-Cre transgenic mice. These data are representative from three independent experiments. (b) LRP2 mRNA levels in metabolic organs in LRP2$^{loxP/loxP}$ (control) and M-LRP2$^{-/-}$ mice. LRP2 mRNA was measured by qPCR (BAT, WAT, pancreas, kidney: n = 2 per group, liver: n = 3 for per group, muscle: n = 5 for control, n = 3 for M-LRP2$^{-/-}$, heart: n = 3 for control, n = 2 for M-LRP2$^{-/-}$). (c) Quantification of insulin signaling components from Fig. 5j in control and M-LRP2$^{-/-}$ mice fed a chow diet (n = 3 per group). (d) LRP2 mRNA levels in C2C12-myc-GLUT4 muscle cells. Cells were transfected by control siRNA or LRP2 siRNA. LRP2 mRNA levels were measured by qPCR from 3 independent experiments. All bars and errors represent means ± SEM. $^*p < 0.05$, $^{**}p < 0.01$ vs. control by two-sided Student's t test. (e) LRP2 expression in muscle of LRP2$^{loxP/loxP}$ (control) and M-LRP2$^{-/-}$ mice. In situ proximity-ligation assay (PLA) was performed. This assay can detect one single protein with high sensitivity when only one primary antibody is used. Muscle section was incubated with primary antibody against LRP2. Red spot bulbs indicate the LRP2 protein. The nuclei were stained with DAPI (blue). The scale bars represent 25 µm.

(f) Colocalization of endogenous ApoJ and LRP2 in muscle of LRP2$^{loxP/loxP}$(control) and M-LRP2$^{-/-}$ mice. In situ proximity-ligation assay (O=link) was performed. Cells were incubated with primary antibodies against ApoJ and LRP2 overnight. Red spot bulbs indicate the interaction with endogenous ApoJ and LRP2. The nuclei were stained with DAPI (blue). Arrows indicates colocalization of endogenous ApoJ and LRP2. The scale bars represent 25 µ m. (g) ApoJ binding to LRP2 in C2C12-myc-GLUT4 muscle cells. Cells were treated with 100 nM ApoJ or 100 nM insulin for 15 min. In situ proximity-ligation assay (O=link) was performed. Red spot bulbs indicate the interaction with exogenous ApoJ and LRP2. The nuclei were stained with DAPI (blue). The scale bars represent 50 µm. Data for e-g are representative from three independent experiments.

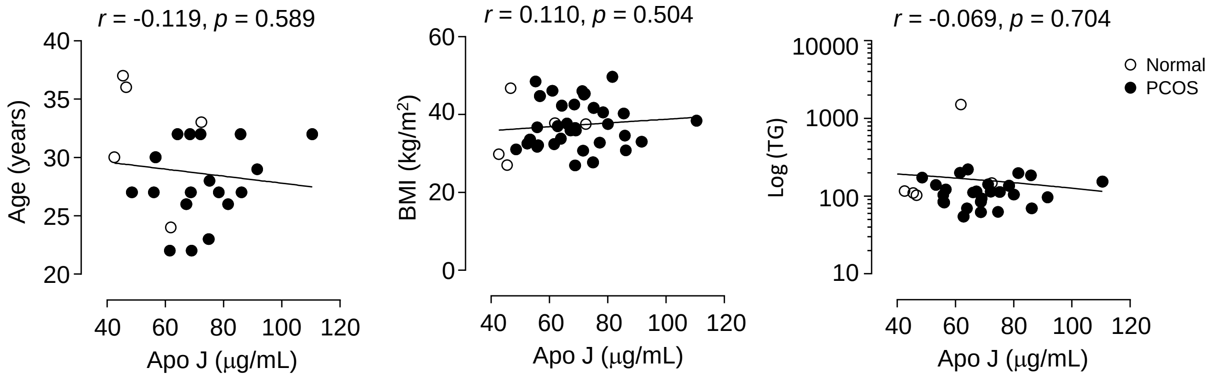

Supplementary Fig. 5 Relationship of serum ApoJ with age, BMI, and TG levels in normal cycle and PCOS subjects. p values were obtained by Spearman's rank correlation analysis and r values indicate Spearman's correlation coefficient.
