# Supplementary material for: Apolipoprotein J is a hepatokine regulating muscle glucose metabolism and insulin sensitivity
Source: Nat Commun. 2020 Apr 24;11:2024. doi: 10.1038/s41467-020-15963-w (PMC7181874; doi:10.1038/s41467-020-15963-w)
Supplement: Supplementary file 3 — Reporting Summary [file 41467_2020_15963_MOESM3_ESM.pdf]

natureresearch

Corresponding author(s): Young-Bum Kim

Last updated by author(s): Mar 8, 2020

Reporting Summary

Nature Research wishes to improve the reproducibility of the work that we publish. This form provides structure for consistency and transparency in reporting. For further information on Nature Research policies, see Authors & Referees and the Editorial Policy Checklist .

Statistics

For all statistical analyses, confirm that the following items are present in the figure legend, table legend, main text, or Methods section.

n/a	Confirmed	
☐	☒	The exact sample size (*n*) for each experimental group/condition, given as a discrete number and unit of measurement
☐	☒	A statement on whether measurements were taken from distinct samples or whether the same sample was measured repeatedly
☐	☒	The statistical test(s) used AND whether they are one- or two-sided<br>*Only common tests should be described solely by name; describe more complex techniques in the Methods section.*
☐	☒	A description of all covariates tested
☐	☒	A description of any assumptions or corrections, such as tests of normality and adjustment for multiple comparisons
☐	☒	A full description of the statistical parameters including central tendency (e.g. means) or other basic estimates (e.g. regression coefficient) AND variation (e.g. standard deviation) or associated estimates of uncertainty (e.g. confidence intervals)
☐	☒	For null hypothesis testing, the test statistic (e.g. *F*, *t*, *r*) with confidence intervals, effect sizes, degrees of freedom and *P* value noted<br>*Give P values as exact values whenever suitable.*
☒	☐	For Bayesian analysis, information on the choice of priors and Markov chain Monte Carlo settings
☒	☐	For hierarchical and complex designs, identification of the appropriate level for tests and full reporting of outcomes
☒	☐	Estimates of effect sizes (e.g. Cohen's *d*, Pearson's *r*), indicating how they were calculated

Our web collection on statistics for biologists contains articles on many of the points above.

Software and code

Policy information about availability of computer code

Data collection	Images data were collected by AxioVision software (v 4.2 SP1).
Data analysis	Insulin signaling quantitation data were analyzed by Image J (v1.52a, NIH). Images were analyzed by AxioVision software (v 4.2 SP1). Data analysis for statistical significance were performed using Stat View (v.5.0) or SPSS (v 18.0).

For manuscripts utilizing custom algorithms or software that are central to the research but not yet described in published literature, software must be made available to editors/reviewers. We strongly encourage code deposition in a community repository (e.g. GitHub). See the Nature Research guidelines for submitting code & software for further information.

Data

Policy information about availability of data

All manuscripts must include a data availability statement. This statement should provide the following information, where applicable:

- Accession codes, unique identifiers, or web links for publicly available datasets
- A list of figures that have associated raw data
- A description of any restrictions on data availability

Source data for the figures and table are provided with the paper. Source data underlying Figs. 4a–j, 4l–o, 5a–b, 5d–l, 6a–b, 7a–d, and 8a–l, and Supplementary Figs. 2a–l, 3a–h, 4b–d, and 5 are provided as a Source Data File, The numerical data underlying Table 1 is provided as a Source Data File.

Field-specific reporting

Please select the one below that is the best fit for your research. If you are not sure, read the appropriate sections before making your selection.

Life sciences study design

All studies must disclose on these points even when the disclosure is negative.

Sample size	Sample size was determined by our experience with inherent variability, especially with outbred strains. No statistical method was used to predetermine sample size.
Data exclusions	Data were only excluded for failed experiment, including insulin or glucose injection experiment.
Replication	Each experiments presented in our manuscript was repeated in multiple mice. Replication experiments for animal study were successful. For cell culture experiments, each experiment was repeated in 2-3 times with the same condition independently. All information was described in method section and figure legends. Replication experiments were successful.
Randomization	All mice used in all experiments of the current study were randomly grouped for control and knockout mice.
Blinding	Blood parameter measurements were performed blinded. The investigator had no knowledge of the genotype at that point in time. This is stated in Method section under Blood parameter measurements.<br>All laboratory determinations in materials from human subjects were performed on deidentified samples. Investigators were blinded to group allocation until final data analysis. This is stated in Method section under Clinical Study.

Reporting for specific materials, systems and methods

We require information from authors about some types of materials, experimental systems and methods used in many studies. Here, indicate whether each material, system or method listed is relevant to your study. If you are not sure if a list item applies to your research, read the appropriate section before selecting a response.

Materials & experimental systems

n/a	Involved in the study	
☐	☒	Antibodies
☐	☒	Eukaryotic cell lines
☒	☐	Palaeontology
☐	☒	Animals and other organisms
☐	☒	Human research participants
☐	☒	Clinical data

Methods

n/a	Involved in the study	
☒	☐	ChIP-seq
☒	☐	Flow cytometry
☒	☐	MRI-based neuroimaging

Antibodies

Antibodies used	The information of all antibodies, including catalog number, the name of company, supplementary information, are described in method section. Polyclonal antibodies against phosphor-Y972 IR (Cat#: 44-800G); phosphor-Y612 IRS-1 (Cat#: 44-816G) (both Invitrogen; Carlsbad, CA); phospho-Ser473Akt (Cat#: 4060); phospho-Thr308Akt (Cat#: 4056); phospho-Thr642AS160 (Cat#: 4288); phospho-Ser21/9 GSK3 (Cat#: 9331); phospho-Ser641glycogen synthase (GS) (Cat#: 3891); phosphor-Ser240/244 S6 ribosomal protein (Cat#: 2215) (all Cell Signaling Technology, Beverly, MA); active MAPK (pTEpY) antibody (Cat#; V8031); Promega, Madison, WI); ApoJ/Clusterin (sc-8354); IR (sc-711); Akt (sc-7126) ; GAPDH (sc-47724); selenoprotein P (sc-376858) (all Santa Cruz Biotechnology, Dallas, TX) or monoclonal antibodies against ß-actin (A2228, Sigma-Aldrich, St. Louis, MO).Dilution of all antibodies was 1:1000 except active MAPK, which was 1:5000.
Validation	All of the antibodies used in this study have been previously published and their specificity has been validated either by us or others (Nat Comm 2013, 4:1862, JBC 2009, 284:1176-11780, JBC 2000, 275: 18318-18326, JBC. 2007, 23829-23840, Cell Metabolism 2010, 12: 483-495, JBC 2014, 289: 1246-84).<br>p-Y972 IR antibody: Invitrogen, Cat# ;44-800G; Application/Dilution; WB/1:1000<br>p-Y612 IRS-1 antibody: Invitrogen, Cat#; 44-816G; Application/Dilution; WB/1:1000<br>p-Ser473Akt antibody: Cell Signaling Technology, Cat#: 4060; Application/Dilution; WB/1:1000<br>p-Thr308Akt: Company: Cell Signaling Technology, Cat#: 4056; Application/Dilution; WB/1:1000<br>p-Thr642AS160: Company: Cell Signaling Technology, Cat#; 4288; Application/Dilution; WB/1:1000<br>p-Ser21/9 GSK3: Company: Cell Signaling Technology, Cat#; 9331; Application/Dilution; WB/1:1000<br>p-Ser641glycogen synthase9331 (GS): Cell Signaling Technology, Cat#; 3891; Application/Dilution; WB/1:1000<br>p-Ser240/244 S6 ribosomal protein: Cell Signaling Technology, Cat#; 2215; Application/Dilution; WB/1:1000<br>active MAPK (pTEpY) antibody: Promega, Cat#; V8031; Application/Dilution; WB/1:5000<br>ApoJ/Clusterin antibody: Santa Cruz, Cat#; sc-8354; Application/Dilution; WB/1:1000, PLA/1:200(for tissue), 1:100 (cell)<br>IR antibody: Santa Cruz, Cat#; sc-711; Application/Dilution; WB/1:1000<br>Akt antibody: Santa Cruz, Cat#; sc-7126; Application/Dilution; WB/1:1000<br>GAPDH antibody: Santa Cruz, Cat#; sc-47724; Application/Dilution; WB/1:1000<br>selenoprotein P antibody: Santa Cruz, Cat#; sc-376858; Application/Dilution; WB/1:1000

ß-actin antibody: Sigma-Aldrich, Cat#; A2228; Application/Dilution; WB/1:1000
LRP2 antibody: Novus biologicals, Cat#; NB110-96417, Application/Dilution; PLA/1:200(for tissue), 1:100 (cell)
FOXO1 antibody: Abcam, Cat#; ab70382; Application/Dilution; IF/:50
IgG antibody: Invitrogen, Cat#; A-11008; Application/Dilution; IF/1:500

Eukaryotic cell lines

Policy information about cell lines

Cell line source(s)
C2C12 cells are obtained from ATCC (CRL-1772). C2C12-myc-GLUT4 cells are from Dr. Amira Klip (The Hospital for Sick Children, Toronto, Canada

Authentication
Cell lines have been authenticated by light microscopy.

Mycoplasma contamination
Cell lines were tested monthly for mycoplasma contamination. We confirmed that all cell lines tested negative for mycoplasma contamination.

Commonly misidentified lines (See ICLAC register)
No commonly misidentified lines were used in the study.

Animals and other organisms

Policy information about studies involving animals; ARRIVE guidelines recommended for reporting animal research

Laboratory animals
We provided animal species, strain, sex and age of animals in method and individual figure legends.Animals bearing a LoxP-flanked ApoJ allele (ApoJloxP/loxP mice) were generated by inGenious Targeting Laboratory (Stony Brook, NY). Briefly, a BAC clone (C57BL/6, RPC123 clone) containing a 9.32kb fragment of ApoJ genomic DNA was used to generate a targeting vector. Four independent ApoJloxP/+ ES clones were identified, which were injected into C57BL/6 blastocysts to generate chimeric mice. The chimeric mice were bred with wild type C57BL/6 mice for germline transmission. Heterozygous animals were then crossed with mice expressing flp-recombinase in the germline (Flipper mice, from The Jackson Laboratory, Bar Harbor, ME) to delete the FRT-flanked Neo cassette. Offspring of these mice were heterozygous for the desired ApoJloxP/+ allele. Albumin-Cre; ApoJloxP/loxP mice (liver-specific ApoJ-deficient mice; L-ApoJ-/-) and myogenin-Cre; ApoJ loxP/loxP (muscle-specific ApoJ-deficient mice; M-ApoJ-/-) were generated by mating ApoJ loxP/loxP mice with albumin-Cre or myogenin-Cre transgenic mice, respectively. For generation of muscle-specific LRP2-deficient mice (M-LRP2-/-), LRP2 loxP/loxP mice53 were crossed with myogenin-Cre transgenic mice. Genotypes of these mice were identified by polymerase chain reaction (PCR) or immunoblotting. Myogenic-Cre transgenic mice were generously provided by Dr. Zoltan Arany (University of Pennsylvania, Philadelphia, PA). Albumin-Cre transgenic mice and global ApoJ-deficient min (ApoJ-/-) were purchased from The Jackson Laboratory (Bar Harbor, Maine).

The mice were fed standard chow (Teklad F6 Rodent Diet 8664, Harlan Teklad, Indianapolis, IN) or a high-fat diet (HFD) with 58% kcal in fat (D12331, Research Diets, New Brunswick, NJ) and housed under controlled temperature at 22–24°C and a 12 h light/12 h dark cycle, and the humidity is between 40-60%.

Wild animals
The study didn't involve wild animal.

Field-collected samples
The study didn't involve samples collected from the field.

Ethics oversight
Animal studies were conducted in accordance with the National Institutes of Health "Guide for the Care and Use of Laboratory Animals" (NIH Publication No. 85-23, revised 1996) and approved by the Institutional Animal Care and Use Committees of Beth Israel Deaconess Medical Center.

Note that full information on the approval of the study protocol must also be provided in the manuscript.

Human research participants

Policy information about studies involving human research participants

Population characteristics
28 female participants with a diagnosis of polycystic ovary syndrome (based on the 1990 National Institutes of Health Conference criteria for PCOS), were recruited and randomized to placebo or pioglitazone 45 mg daily.. Average age was 27.87 years, average BMI 36.29 kg/m2, average fasting glucose 93.77 mg/dl, average 2-hour post-glucose challenge glucose 150 mg/dl. Six normal control subjects were included for comparison (average age 31.83, BMI 35.93, fasting glucose 97.70 mg/dl, 2-hour glucose 123.5 mg/dl).

Recruitment
Participants were recruited from the community (flyers, advertisements, local PCOS support group), and from clinics and physicians within the VA/UCSD healthcare systems.1. We are not aware of any biases, self-selecting or otherwise in the study design. One potential limiting factor was that if they were taking oral contraceptives, subjects had to agree to stop their use for at least 2 months before screening and throughout the study period. (Don't think this made any difference, but can't say for sure if any one failed screening by refusing to stop OCPs.)

Ethics oversight
The project was approved by the Institutional Review Boards of the Veterans Affairs San Diego Healthcare System and the University of California, San Diego

Note that full information on the approval of the study protocol must also be provided in the manuscript.

Clinical data

Policy information about clinical studies

All manuscripts should comply with the ICMJE guidelines for publication of clinical research and a completed CONSORT checklist must be included with all submissions.

Clinical trial registration	The study was initiated before ClincalTrials.gov registration was required.
Study protocol	Full protocol is stored on the restricted access Research drive of the VA San Diego Healthcare System.
Data collection	Data were collected at the VA San Diego Special Diagnostics and Treatment Unit and the UCSD General Clinical Research Center. First subject was enrolled on 1/18/2002. Last clinical data was collected on 01/06/2006.
Outcomes	Primary outcome measures included whole body insulin action as measured by hyperinsulinemic euglycemic clamp and ovarian androgen biosynthesis as measured by leuprolide-stimulated production of 17-hydroxyprogresterone. There were no formal pre-defined secondary outcome measures in the original clinical study. Subsequently, a number of measures were made using existing samples (see Ciaraldi TP, et al. Metabolism 62:1587, 2013; do:10.1016/j.metabol.2013.07.004), but the study was not powered for those measures. Circulating factors were assessed by ELISA and Millex Multiplex assays, as detailed in the publication cited above.
